# Metabolomics and Human Health: Progress, Insights, Challenges and the Concept of a Healthy Metabolome

**DOI:** 10.3390/biom16050683

**Published:** 2026-05-05

**Authors:** Lin Fang, In Soo Kim, Brendan R. Martin, Lee Sherlock, Soyoung Min, Young Kook Kim, K. H. Mok

**Affiliations:** 1 Trinity Biomedical Sciences Institute (TBSI), School of Biochemistry & Immunology, Trinity College Dublin, The University of Dublin, Dublin 2, D02 PN40 Dublin, Ireland; fangl@tcd.ie (L.F.); mins@tcd.ie (S.M.); 2 Department of Pharmacology, Chungnam National University College of Medicine and Biomedical Research Institute, Chungnam National University Hospital, Daejeon 35015, Republic of Korea; insk@cnu.ac.kr; 3 Department of Medical Science, Chungnam National University College of Medicine and Biomedical Research Institute, Chungnam National University Hospital, Daejeon 35015, Republic of Korea; 4 Meta-Flux Ltd., Dublin 1, D01 Y6H7 Dublin, Ireland; brendanm@meta-flux.com (B.R.M.); leesherlock@meta-flux.com (L.S.); 5 Department of Ophthalmology, Seoul National University Hospital, Seoul National University College of Medicine, Seoul 03080, Republic of Korea; eyedry@snu.ac.kr

**Keywords:** metabolomics, metabolic baseline, mass spectrometry, NMR spectroscopy, population cohorts, longitudinal studies, physiological homeostasis

## Abstract

Health is increasingly recognized as a dynamic state of physiological equilibrium rather than the mere absence of disease. Traditional clinical biomarkers capture only limited aspects of physiology and fail to reflect the multidimensional and dynamic nature of human homeostasis. Metabolomics, by comprehensively profiling small-molecule metabolites downstream of genetic, proteomic, environmental, and lifestyle influences, offers a sensitive and functional readout of an individual’s physiological state. This review catalogues current advances in applying metabolomics to characterize metabolic features of health, focusing on the influence of age, sex, body mass index, physical activity, diet, lifestyle behaviors, microbiome composition, and population heterogeneity. Numerous cohort studies have shown that substantial metabolic variability exists amongst individuals within apparent healthy populations, underscoring the need for stratified and contextual reference frameworks. We further discuss major challenges in defining a standardized metabolic baseline, including analytical platform heterogeneity, biofluid specificity, population diversity, and the predominance of cross-sectional study designs. Finally, we highlight the role of large-scale longitudinal cohorts, biobanks, multi-omics integration, and artificial intelligence–driven tools in overcoming these barriers. Establishing robust, dynamic, and personalized metabolic baselines will be critical for redefining health, enabling early intervention, and supporting predictive and preventive medicine.

## 1. Introduction

The World Health Organization (WHO) describes health as “a state of complete physical, mental, and social well-being and not merely the absence of disease or infirmity” [1]. However, the human body’s physiological state is not static but exhibits complex dynamic characteristics [2]. Consequently, there is an increasingly urgent need to establish a biochemical standard reference system—the molecular baseline of health—that reflects the body’s physiological equilibrium. Defining a health baseline informs precision medicine (disease prevention and early detection based on individual risk) [3] and population health (health outcomes of a group of individuals) [4]. It not only helps researchers identify subtle physiological changes before clinical symptoms emerge but also provides scientific grounds for personalized prevention strategies [5].

Currently, many traditional clinical biomarkers measure only single dimensions, such as blood glucose, cholesterol, liver enzymes, or inflammatory proteins like C-reactive protein. These reflect only partial aspects of human physiological states [6], whereas the body’s physiological systems are inherently dynamic and multidimensional. Physiological homeostasis is a dynamic equilibrium maintained by interconnected molecular systems [2]. Even in the absence of clinical symptoms, subtle imbalances at the molecular level may already signal early deviations from health [7]. For example, biomedical research has identified a range of physiological states that precede the onset of overt disease, such as: subclinical inflammation [8], pre-metabolic syndrome [9] and functional decline prior to frailty [10]. Therefore, developing a molecular framework that comprehensively represents the human body’s biochemical state remains a formidable challenge in the biomedical field [11,12,13].

Metabolomics is a comprehensive approach to measure the concentration and distribution of various metabolites (e.g., sugars, lipids, amino acids, and other small molecules <10 kda) in human samples (such as blood, urine, cerebrospinal fluid, and saliva) using mass spectrometry (MS) or nuclear magnetic resonance spectroscopy (NMR) in response to the expression of genes and proteins, and to correlate these results with known physiologic or pathologic conditions [14,15,16,17].

**Figure 1:**
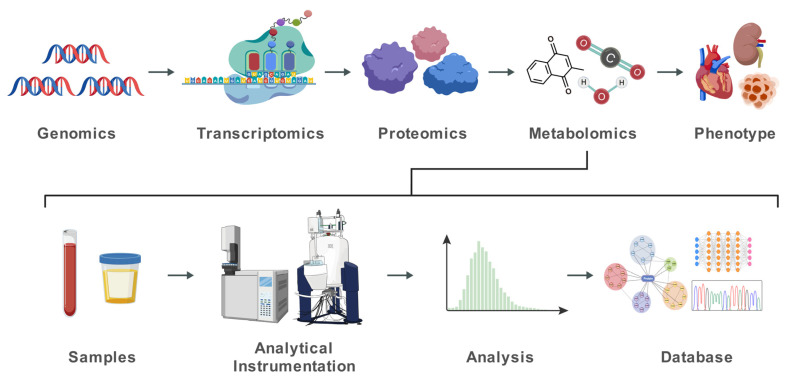
Biological samples are detected, analyzed and characterized metabolically to form a bridge between the genome and the phenotype to provide a more comprehensive understanding of biological systems. Created with BioGDP.com [39].

Compared to the other Omics technologies, metabolomics provides unique advantages. Since the concentrations of cell metabolite products are constantly in flux, metabolomics can directly reflect the functional state of cellular activity with great sensitivity [18]. While genomics reveals genetic potential and proteomics identifies protein expression [19], metabolomics can show a unique metabolic readout of the health or disease status of the downstream biochemical consequences of genetic and environmental influences [20]. Metabolomics is highly sensitive to various genetic and environmental stimuli, playing an important role in the early diagnosis of diseases or the monitoring of subtle changes before diseases occur [21] and has been widely applied in the research of metabolic pathway disorders in various disease types, such as diabetes [22,23,24,25], cardiovascular diseases [26,27,28,29], congenital metabolic defects [30,31,32], and various cancers [33,34,35,36,37,38]. In addition, metabolomics is very closely related to the phenotype of organisms, responding to the influence of environmental factors, genetic causes, and disease conditions on phenotype, and thus bridges the gap between genotype and phenotype and complements genomic and proteomic data to provide a more comprehensive understanding of biological systems [18] (Figure 1).

A fundamental question arises: if we can detect imbalance, what constitutes the objective definition of balance? To explain metabolic imbalance at the molecular level in the human body, one must first establish a clear “health baseline.” Without standardized reference criteria, it becomes difficult to determine whether the metabolic changes detected represent normal individual variation or early signs of disease. It follows that defining a standard health baseline requires a comprehensive capture of the diversity of human health, therefore necessitating large-scale population studies, standardized analytical methods, and integration of multi-omics data.

To this end, the Project Baseline Health Study (PBHS) serves as a notable example [5]. The objectives of the study are “(i) to develop the requisite tools and technologies, (ii) evaluate the use of sensor technologies, (iii) create a dataset encompassing a wide spectrum of phenotypic measures, (iv) measure the phenotypic diversity of the participants, and (v) share this vast data and create an example of open science” [40]. Currently, this research has been applied to various fields, such as pre-diabetes, epigenetic aging, and chronic obstructive pulmonary disease (COPD) [41,42,43]. Data collection is very comprehensive, spanning self-reporting, general practitioner medical, molecular, imaging, sensor-based, behavioral and psychological measurements. For the molecular measurements, lipids, high-sensitivity C-reactive protein (hsCRP), creatinine, ions, serum proteins, glomerular filtration rate (GFR), hematology, thyroid-stimulating hormone (TSH) and others are covered, Although certainly encompassing data from all individuals (healthy and unhealthy), studies such as the systematic integration of metabolic profiles in a healthy state are not specified as its objectives, and therefore the project does not directly answer the question that we have stated above.

This review aims to discuss the role of metabolomics in possibly defining a health baseline by primarily addressing the following aspects: current advances in metabolomics within health research; existing challenges; ongoing large-scale projects and research initiatives worldwide; and prospects.

## 2. Determinants of the Human Metabolomic Baseline

Metabolomics provides a means of characterizing metabolic differences between groups and individuals, while expanding understanding of the biology and biological mechanisms underlying these differences. Metabolomics is influenced by a variety of factors, with age and gender being the two most strongly correlated with metabolomics significance, and these two factors are significantly different in many metabolic pathways [44].

In 2023, by comparing three age groups, Giesbertz et al. found that energy metabolism and oxidative pathways may be altered during the aging process. For example, in correlation with aging, acylcarnitine levels were found to increase [45]. In addition, older adults, especially older women, exhibit an increased proteolytic state and a diminished anabolic response of muscle to insulin and amino acid stimulation, which may be related to anabolic resistance [45]. In 2024, by analyzing plasma metabolites from 2764 people aged 24–110 years, Sebastiani et al. found that 308 metabolites were significantly correlated with age, with lipids (e.g., elevated triglycerides, decreased sphingolipids) and amino acids (e.g., decreased tryptophan) being particularly prominent, of which were then validated through an 8-year follow-up study [46]. The studies conducted by Giesbertz et al. and Sebastiani et al. were both cross-sectional in design, comparing metabolite profiles among individuals of different age groups at a single time point. Moreover, when participants were stratified by waist circumference, the direction of change for certain amino acids and acylcarnitines shifted significantly. Such results suggest that apart from age, other factors such as insulin resistance, kidney function, medication use and degree of obesity, may be affecting the metabolic level. Consequently, some metabolic alterations attributed to “age differences” may in fact be partially driven by these confounding variables.

Identification of the male/female trait through metabolomics had also been clearly demonstrated in the KarMeN (Karlsruhe Metabolomics and Nutrition) study (2017), where urine and plasma samples from 301 healthy participants were analyzed using a multi-platform approach [47,48]. The research found that the accuracy rate of predicting gender based on the metabolite spectrum was very high, reaching 95% and 90% for plasma and urine samples, respectively. In the plasma samples, females showed higher levels of creatine, phosphate, glycine, sphingomyelin C18:1 and several phosphatidylcholines, whereas males exhibited higher concentrations of branched-chain amino acids (leucine, isoleucine, and valine), creatinine, and uric acid. Similar trends were also observed in the urine samples: males have higher concentrations of creatinine and certain organic acids, while females have higher concentrations of citrate and phenolic metabolites. The authors further suggested that sex hormones account for the observed metabolic differences between men and women. Collectively, these findings demonstrate that sex is a major determinant of metabolic individuality, influencing amino acid, lipid, and energy metabolism across populations. Another study, the Tohoku Medical Megabank Cohort Study (2024) analyzed plasma metabolites in 39,239 healthy participants and found significant gender differences in metabolite concentrations across all the team’s studies. Branched-chain amino acid (BCAA) levels were generally higher in the male population, while dramatic fluctuations in metabolites such as glutamine and carnitine were seen in the female population during the perimenopausal period (45–54 years) [49]. In addition, this study suggests that gender, age and body mass index (BMI) systematically influence one another, providing a key basis for gender-specific health management [49].

Notably, although the two studies were conducted on different populations and employed distinct analytical platforms, they nonetheless yielded largely consistent results regarding sex-related metabolic differences. Men generally exhibited stronger protein and energy metabolism, whereas women showed differences in lipid utilization and organic acid metabolism. This cross-population and cross-regional consistency indicates that sex-dependent metabolic variation represents a fundamental physiological characteristic of humans rather than a culture- or diet-specific phenomenon. It should be noted the KarMeN study (i) included only 301 participants and therefore lacked sufficient representation and (ii) the average age of the subjects were rather high (44.4 for males, 51.7 for females), while (iii) the Japanese cohort, despite its large sample size, did not adequately control for key variables such as hormonal status, menstrual cycle, and lifestyle factors, all of which may influence metabolic outcomes.

In addition to gender and age, many acquired factors are also closely related to human metabolites. Firstly, changes in BMI alone have a big impact on metabolites. Cirulliet et al. and Zhong et al. both investigated the influence of BMI on the human metabolome in healthy adult populations and, despite employing different analytical platforms, reached highly consistent conclusions [50,51]. With increasing BMI, the levels of branched-chain amino acids, lipid metabolic intermediates, and triglyceride-rich lipoproteins were elevated, while polyunsaturated fatty acids (PUFAs) and high-density lipoprotein (HDL) levels decreased. This indicates that even within healthy populations, metabolomics can detect subtle biochemical differences, whereas BMI can only account for a portion of the diversity underlying metabolic health [52]. What truly needs to be explored is the molecular-level metabolic homeostasis that underpins these variations.

Physical activity (PA) is a globally recognized determinant of metabolic health [53], influencing lipid, amino acid, and energy metabolism, and here also, metabolomics provides a powerful tool to capture these molecular changes, offering deeper insights into how regular exercise helps maintain physiological homeostasis [15]. By studying 7271 diabetes-free men in Finland, a total of 198 metabolites could be associated with PA. For example, in one of the high physical activity groups, the analyses showed a trend towards a positive correlation between choline plasmalogens and PUFAs, with the opposite result for bile acids and short chain acylcarnitines. Many of the metabolites identified were also significantly associated with insulin sensitivity and Type 2 Diabetes (T2D) risk [54]. These findings suggest that regular physical activity promotes favorable lipid and amino acid metabolism, contributing to improved metabolic flexibility. In addition, one special population related to physical activity consists of individuals engaged in high-intensity exercise, such as athletes. This group generally represents a highly healthy population; therefore, some researchers have used athletes as study subjects to explore their characteristic metabolic profiles. Parstorfer et al. found that endurance athletes exhibited lower levels of lipids, including triglycerides, diglycerides, as well as reduced concentrations of glutamate and arginine, indicating higher fatty acid oxidation capacity and improved insulin sensitivity. In contrast, strength-trained athletes showed elevated levels of free carnitine (C0), 3-methylhistidine (3-Met-His) and gamma-aminobutyric acid (GABA), suggesting enhanced mitochondrial energy supply and increased protein turnover activity [55]. Such metabolomic evidence helps delineate how physical activity contributes to the molecular baseline of health. Similar to the above studies, most metabolomics studies on physical activity were cross-sectional, limiting the ability to infer causality. Although studies involving athletes provide valuable insights into metabolic adaptations under high-intensity exercise conditions, such findings are difficult to be extended to the broader healthy population. Future research should systematically control and stratify external factors such as physical activity levels to better delineate their specific impact on metabolic homeostasis to establish a more comprehensive molecular baseline of health.

In addition to the human subjects themselves, the microbiome of the host’s intestinal tract has been shown to be important to the metabolic profile of the human body, influencing various aspects such as cardiovascular, neurological, and metabolic health [56,57]. The metabolome in the intestine metabolizes dietary substances to produce a wide range of metabolites, including short-chain fatty acids, trimethylamine-N-oxide (TMAO), and a series of tryptophan breakdown metabolites [58,59,60,61]. As early as 2016, Zhernakova et al. discovered in the LifeLines-DEEP cohort from the Netherlands that 18.7% of the differences in the composition of the human intestinal microbiome could be explained by 126 tested factors (including diet, medication, lifestyle, and clinical indicators), and these differences were further reflected in changes in various types of metabolites such as lipids and amino acids [62]. In addition, Kurilshikov et al. conducted a systematic analysis of the relationship between gut microbiota and plasma NMR metabolome in the population, finding that gut microbiota could explain a portion of the variation in plasma metabolites (up to approximately 11% in the general population and up to approximately 16% in obese individuals), and this influence was more significant in the obese state. The study further indicated that microbial functional pathways are closely related to the composition of lipoprotein subtypes and cardiovascular metabolic risks. Several bacterial metabolic pathways were significantly associated with the cardiovascular disease metabolic risk score constructed based on plasma metabolites, and to a certain extent, they were independent of dietary factors [63]. Visconti et al. used the TwinsUK twin cohort and selected 1004 twins for in-depth research. After controlling for the influence of host genetic confounding factors, the metabolic functions of the intestinal microbiome still had significant correlations with 34% of blood metabolites and 95% of fecal metabolites, totaling over 18,000 associations [64]. These results indicate extensive links between gut microbial metabolic potential and both systemic and faecal metabolic profiles, consistent with evidence that non-genetic/environmental factors play a major role in shaping the gut microbiome, as reported by Rothschild et al. [65].

Similarly, tears are an accessible biofluid that can complement blood and urine for defining metabolic baselines, particularly for longitudinal within-person sampling in outpatient settings [5]. Given that baselines are dynamic, repeated tear sampling may help characterize intra-individual variability and identify early deviations. The ocular surface is a host-microbe-environment interface where local immunity and low-biomass microbial communities can influence biochemical composition. Integrating ocular surface microbiome profiling with tear metabolomics may provide a functional readout of local homeostasis and support compartment-aware baseline models [66,67,68,69,70].

Daily habits such as diet, smoking and alcohol intake also have an impact on metabolites. Rebholz et al. compared a study group using the Dietary Approaches to Stop Hypertension (DASH) diet with a randomized control group and found a significant association between DASH and specific metabolic profiles. The researchers determined that a combination of 10 metabolites, including stachydrine, N-methylproline and β-cryptoxanthin, were effective in differentiating the randomized control group from the DASH group [71]. In addition, Li et al. analyzed by metabolomics that chronic alcohol consumption also led to changes in nearly 60 metabolites, such as phosphatidylcholine PC 32:1 and cholesteryl ester CE 16:1, highlighting the significant metabolomic impact of chronic alcohol intake on the human metabolome [72]. Aslam et al. utilized LC-MS/MS to compare the systemic metabolic differences between smokers and non-smokers, identifying 930 metabolites, among which 343 were significantly altered between the two groups. After screening based on fold change (±1.5), it showed that 243 metabolites were dysregulated (116 upregulated and 127 downregulated). The differential signals mainly pointed to pathways related to amino acids and nucleotide/sugar metabolism, and exhibited characteristics of oxidative stress, such as a significant decrease in biliverdin and an increase in oxidized glutathione; at the same time, higher blood cadmium levels were detected in smokers and a negative correlation was found with blood zinc. In addition to the changes in metabolites, smokers also showed significant abnormalities in blood glucose indicators, lipid profiles, and inflammatory indicators (such as CRP, IL-6), which were consistent with the systemic metabolic disturbances revealed by the metabolomics [73].

The studies mentioned above generally involved analyzing populations for specific lifestyles alone and exploring their effects on human metabolomics. In contrast, there are currently only a handful of studies at present that seek to find the relationship between metabolomics and a composite healthy lifestyle score through cohort studies, and from that, derive a baseline to help academics define human health. Tessier et al. identified unique metabolomic profiles associated with a healthy lifestyle (healthy diet, moderate alcohol intake, regular physical activity, maintaining a healthy weight, and no smoking) in a prospective study involving 13,056 participants from four cohorts in the United States [73]. Metabolite analysis revealed that cholesterol esters and phosphatidylcholine lipids, primarily in the lipid metabolic pathway, tended to correlate positively with higher healthy lifestyle scores, while short-chain and high-saturated triglycerides and diglycerides correlated negatively. Analysis of more than 4000 deaths recorded over 28 years of follow-up found that participants with higher Healthy Lifestyle Scores had a significantly lower risk of all types of death. This study provides additional useful information for researchers to explore the relationship between a comprehensive healthy lifestyle and specific metabolic pathways [74]. In addition to studying the impact of individual lifestyle factors on human metabolism, researchers have also begun integrating multiple behaviors into a comprehensive healthy lifestyle score to better reflect overall health status. In 2024, Hantikainen et al. conducted a study of the relationship between metabolomics and healthy population status for the Cooperative Health Research in South Tyrol (CHRIS) cohort (3142 healthy adults) [74]. With a unique analytical approach, the researchers identified 21 metabolites outside of age. Of these, elevated glutamate and lowered serotonin levels showed significant associations with disease status independent of age. A variety of metabolites were also identified as being associated with cardiovascular and kidney disease. This study also provides reliable data to support the emergence of biomarkers for predicting health in the future [75].

From these studies outlined above, while individual-level variability (such as age, sex, BMI, diet, and lifestyle) clearly results in significant metabolic differences, the impact brought by the heterogeneity of the cohorts themselves cannot be ignored as well. Due to the influence of various factors such as culture, social environment, economy, and genetics [76,77,78,79], metabolic differences between ethnicities or populations have emerged as important factors that need to be taken into account. Trivedi et al. recently analyzed 572 male individuals from White European (WE), South Asian (SA), and African-Caribbean (AC) ethnic groups in the UK using Liquid Chromatography-Mass Spectrometry (LC-MS) and Gas Chromatography-Mass Spectrometry (GC-MS) and have observed clear ethnic group-specific metabolic characteristics [79]. The accuracies for correct classification were 90.53% and 85.58% respectively, with fatty acids and carboxylic acids, glycerophospholipids, steroids, organic acids, amino acid derivatives found to be the key contributing factors for classification. Using pathway enrichment analysis, it was revealed that significant differences in metabolism (TCA cycle), N-glycans, and unsaturated fatty acid biosynthesis were present between different population groups. Hence, even though people live in similar geographical environments, population-level metabolic differences also exist. Therefore, when exploring and constructing the “healthy baseline metabolic profile”, in addition to considering individual-level population variability, population heterogeneity from different ethnic and culture backgrounds must also be incorporated into the framework.

## 3. Technical and Analytical Challenges in Defining a Metabolic Baseline

Defining a standard baseline for health faces numerous obstacles and challenges further still, as current research primarily focuses on single factors or cross-sectional snapshots of healthy populations. To establish a standardized baseline for health, several key challenges must be addressed.

Apart from the variations within the population and the differences among different ethnic groups, the technical differences in the analytical platforms used for metabolomics will further affect the construction of the “health baseline”, making direct comparisons of the data impossible. Currently, the main analytical techniques in metabolomics are NMR spectroscopy and hyphenated techniques combining column chromatography with mass spectrometry (LC-MS, GC-MS and CE-MS), and their main differences and strengths are given in Table 1. Due to these different platform advantages, data from either cannot be directly substituted for each other. NMR spectroscopy has the advantages of quantitative accuracy, stability, and extremely high reproducibility [80,81,82,83,84,85]. In addition, NMR can perform metabolite imaging and metabolic analysis on living samples [82,86,87], and sample processing is relatively simple without complex pretreatment. Since NMR is a non-invasive technique, the samples can also be recovered and used for subsequent experiments [82,83]. Importantly, high-sensitivity NMR spectroscopy (with magnetic fields greater than 16 T and cold probes) can detect metabolites as low as single-digit μM concentrations [88], and the commonly used 1D ^1^H NMR can provide the absolute concentrations of tens to hundreds of metabolites in metabolomics samples [84,89]. However, up to now, MS remains the mainstream method for metabolomics analysis. More than 80% of the published metabolomics studies have adopted MS [82], which is attributed to its high sensitivity, capable of detecting metabolites at concentrations ranging from picomoles (pM) to femtomoles (fM) [90,91], and the number of metabolites that MS can detect is approximately 1000 compounds. In particular, capillary electrophoresis-mass spectrometry (CE-MS) has emerged as a powerful alternative, covering metabolites of key pathways in the central carbon metabolism as most of them are polar and charged (such as phosphorylated carbohydrates, phosphorylated carboxylic acids, amines, amino acids, and nucleic acids) [92,93]. In contrast, the practical detection limit of NMR is typically in the μM range and can only detect approximately 50–200 types of metabolites [82]. Unlike NMR, due to its technical requirements, MS is destructive to the sample, making it unrecoverable for re-detection. However, depending on the probe used, NMR requires a minimum sample volume of 30–600 µL, while for MS analysis, a few microliters are sufficient [83,94]. Therefore, as commented by Marine et al., none of the current analytical methods can fully capture the metabolome [83]. To standardize the “health baseline” detection platform, the only way is to combine different detection platforms. According to previous studies, the metabolites detected by NMR and MS only have minimal overlaps [95,96]. Combining the two can increase the coverage of metabolites, thereby constructing a more complete and better-standardized health baseline, not only because the combination of NMR and MS expands the types of metabolites being detected but also due to the algorithms to detect unknown metabolites [97]. Currently, classic methods such as SUMMIT MS/NMR and NMR–MS Translator algorithms can identify the metabolites of substances without the need for compound purification [98,99]. In the case of such algorithms and the combination of multiple detection platforms, not only can the accuracy of the metabolic spectrum be improved, but also the construction of the health baseline becomes more complete, reliable, and biologically significant.

In addition, metabolic baselines can also be compartment specific. For example, tears, aqueous humor, and ocular surface samples reflect local microenvironments and may only partially track circulating metabolites. This underscores the need for biofluid-specific reference ranges and systematic metadata. Ocular sampling also has distinct pre-analytical sources of variation, including low sample volume, reflex tearing, topical medications, contact lens use, cosmetic products, diurnal effects, and environmental exposures. Standardized collection protocols and consistent metadata capture are therefore prerequisites for robust cross-cohort comparisons and longitudinal inference.

With the development and innovation of artificial intelligence, AI is destined to play a significant role in defining the limits of health. Classic machine learning models such as Random Forest [101,102], Support Vector Machine [103], and Artificial Neural Networks [104,105] assist researchers in selecting metabolic features and classifying health states [106,107,108,109]. Tools like SIRIUS + CSI:FingerID [110,111], and MS2DeepScore [112], through deep learning and using MS/MS spectral data, have become the powerful in identifying unknown key metabolites. The important role of multi-omics integrated data analysis can be carried out with platforms such as OmicsAnalyst [113], Argonaut [114], GraphOmics [115], allowing the combining of genetic, proteomic, microbiome, single or multiple omics data and metabolite data into a unified prediction framework [116].

In terms of current developments, algorithmic interpretation of metabolomic data is operationalized in clinical workflows with measurable real-world impact. The Collaborative Laboratory Integrated Reports (CLIR) platform uses multivariate pattern recognition to integrate tandem MS profiles with covariate adjustments, replacing traditional cutoff values with continuous disease-likelihood scores. CLIR operationalizes multivariate post-analytical interpretation at scale, producing continuous likelihood scores rather than single cutoffs (e.g., hundreds of millions of post-analytical scores reported). Hall et al. report measurable improvements in screening performance (e.g., improved PPV and reduced false positives) for disorders of propionate, methionine, and cobalamin metabolism when CLIR tools and second-tier testing are used [117]. Similarly, NMR-based lipoprotein profiling (NMR LipoProfile) has received FDA 510(k) clearance for cardiovascular risk assessment, with millions of tests performed [118].

In addition, there are research tools that address the annotation bottleneck. Because the vast majority of small molecules lack experimental reference spectra, machine learning (ML) spectrum-prediction and in silico annotation methods are increasingly necessary to scale identification beyond library matches. Graph transformer architectures (MassFormer) predict MS/MS spectra from molecular structures, enabling in silico library expansion beyond existing reference databases [119]. Complementary approaches using fragmentation tree analysis with ML-predicted molecular fingerprints (SIRIUS/CSI: FingerID) achieve substantially higher identification rates than spectral matching alone on benchmark datasets, without requiring spectral library matches [110,111].

Metabolomic profiles are sensitive to diet, circadian timing, and other short-term physiological states. This real-time-, sensitive- and direct-readout advantage may also lead to confounding data due to lifestyle, diet and short-term physiological variation. For technical variation, QC-anchored ML normalization methods (SERRF, hRUV) demonstrate practical reductions in batch effects [120,121]. SERRF, benchmarked against 15 competing methods across three large studies, reduced technical coefficients of variation to 5% RSD, enabling cross-study comparability [120]. For biological confounding, recent work demonstrates that lifestyle factors can be measured objectively as metabolite biomarkers: ML-validated poly-metabolite scores differentiate diet composition (80% vs. 0% ultra-processed food) within-individuals in randomized crossover trials [122]. ML-estimated circadian phase from plasma metabolomics achieves 0.45–0.60 h accuracy under controlled conditions, enabling temporal standardization in future studies [123]. When lifestyle factors are strongly aligned with outcome (limited positivity) or lie on the causal pathway, statistical adjustment is not identifiable and can remove true biological signal. This is a fundamental constraint in causal inference. TRIPOD+AI and PROBAST+AI provide reporting guardrails for defensible model adjustment and when adjustment risks bias [124,125].

However, a critical factor that cannot be overemphasized is the necessity of longitudinal studies or the long-term monitoring data provided by a large cohort of healthy individuals. Noting that the majority of the research data are from cross-sectional studies which capture a “snapshot” of metabolite information at a certain point in time, the emergence of larger cohorts with study periods spanning decades and generations will bring greater confidence to the analyses. In combination, organ-specific phenotyping can help validate how baseline variability translates to measurable outcomes.

A compelling example of this potential is the eye, which offers a unique and accessible window into the systemic metabolic baseline. Ocular fluids, such as tears and the aqueous humor, are not merely local lubricants but possess complex metabolic profiles that reflect the integrity of the blood-retina barrier and systemic neurovascular coupling [126]. Recent studies indicate that metabolomic alterations in these fluids can mirror systemic physiological states, such as diabetic dysregulation or neurodegenerative changes, often preceding clinical symptoms [127,128]. Therefore, integrating ocular metabolomics into large-scale cohorts could provide a more sensitive, non-invasive approach to defining and monitoring the healthy baseline.

Finally, beyond technical and design-related challenges, defining metabolic health baselines is constrained by fundamental conceptual limitations. Traditional approaches often assume a static notion of “normal” metabolism, whereas metabolic homeostasis is inherently dynamic and continuously shaped by age, lifestyle, environmental exposures, and physiological adaptation. Consequently, a single static baseline may fail to capture meaningful variations in metabolic health across time and contexts.

Furthermore, metabolic profiles are highly context-dependent, reflecting the integrated effects of multiple interacting determinants rather than isolated factors. This context dependency complicates efforts to define universal reference ranges applicable across life stages and populations. Defining “healthy” metabolism is particularly challenging across the life course, as metabolic requirements and normative ranges differ substantially between childhood, adulthood, aging, and transitional states such as pregnancy or menopause.

Collectively, these limitations highlight that defining metabolic health baselines is constrained not only by data availability but also by how measurements are generated, standardized, and interpreted over time, reinforcing the need for dynamic, stratified, and context-aware reference frameworks.

## 4. Global Cohorts and Large-Scale Initiatives for Metabolic Baseline Research

A population cohort that can be used for conducting health baseline studies should possess several important characteristics: a sufficiently large sample size to observe population-level variability, a combination of multiple races and regions, healthy volunteers or a healthy sub-cohort, and a complete questionnaire survey (such as gender, age, diet, and lifestyle) that can inform and explain known metabolic variations. Additionally, high-quality metabolic data, the combination of multi-omics data, and the ability to conduct longitudinal follow-ups are also required.

Although there are currently no perfect cohorts that fully meet these conditions globally, the following have shown to closely approach these criteria (Supplementary Table S1):

The Tohoku Medical Megabank Project (TMM) cohort study is a community-based prospective cohort study that was established after the 2011 Great East Japan Earthquake. Launched in the areas affected by this catastrophic geographical event, its aims are to support community health, assess the long-term impact of the earthquake on the disaster victims, and study the influence of gene-environment interactions on the incidence of major diseases such as cancer and cardiovascular diseases [129,130,131,132]. It has also established comprehensive biomedical resources for Japan’s precision medicine [133]. Currently, TMM has recruited 150,000 adult men and women aged 20 and above to participate in the project [133,134,135]. In 2020, Koshiba et al. conducted an analysis and study on 1008 Japanese individuals in the TMM, identifying multiple genetic loci related to circulating metabolites (such as lipids, amino acids, and microbiome-related metabolites). While verifying the effects of known gene loci (such as the FADS cluster on lipid metabolism), they also discovered that intestinal microbial metabolites can be regulated by host genes (such as ACSM2A, GLYAT) [136]. Recently, Takase et al. combined genomic and metabolomic data and discovered that 14 circulating metabolites such as glucose, branched-chain amino acids (leucine, valine), glutamate, and 2-hydroxybutyrate, along with glycine, have a positive correlation with the incidence of T2DM. while glycine showed a negative correlation. For the first time, they also identified seven (7) metabolites (3-hydroxyisobutyric acid, 2-aminobutyric acid, 2-ketoinositol acid, 2-hydroxybutyric acid, leucine, glycine, and glucose) that are involved in mediating the association between genetic risk scores (PRS) and the onset of T2DM, revealing the role of genetics in mediating metabolic pathways and thereby influencing the risk of diabetes [137]. TMM not only generated large-scale metabolomics datasets using NMR and LC-MS platforms, providing detailed metabolic profiles for thousands of participants, but also created an extensive genomic information database [49,136,137,138,139]. The high-quality omics data have made TMM one of the most comprehensive resources for characterizing metabolic variations in East Asia and establishing population-specific health reference ranges.

The Cooperative Health Research in South Tyrol (CHRIS) study is a research cohort based on ordinary adults established in the central and upper parts of the Vals region and the Val Venosta area in Italy [140,141,142,143]. The prevalence of chronic diseases in this regional group is relatively low and their health conditions are generally good [144], and its aim is to explore the genetic and molecular basis of common age-related chronic diseases, as well as how genetic, personal lifestyle, and environmental factors affect human susceptibility and resistance to these diseases [145]. Currently, the CHRIS project has recruited over 13,000 community-dwelling adults and collected extensive questionnaires, lifestyle, and clinical information and has reported many related metabolomics, genomics, and proteomics research results [75,146,147,148,149]. In 2022, Verri Hernandes et al. conducted targeted metabolomics analysis on 6872 participants from the CHRIS cohort. They discovered 42 metabolites associated with gender differences (for example, serotonin has a higher content in females): age was associated with 148 metabolites, with most of them increasing with age, while serotonin showed a negative correlation with age. Additionally, based on BMI analysis, branched-chain amino acids, tyrosine, creatinine, and other metabolites were significantly affected [149]. In the same year, König et al. also conducted an Exome-wide association analysis (ExWAS) on the serum metabolites of 5505 volunteers from the CHRIS cohort. Among the 85 genetic variations identified that had significant associations with metabolites, 39 were new associations that had not been reported previously. Additionally, 15 associations at 10 variants that were significantly enriched in the CHRIS population compared to the European reference population were discovered. For instance, pathogenic variations in the ETFDH and MCCC2 genes were respectively associated with the levels of hexanoylcarnitine and hydroxyvalerylcarnitine [148]. It is notable that the prime feature of the CHRIS project is in its longitudinal design, and it will conduct research through repeated in-person study visits, combined with genotypic and multi-omics data [144]. This feature enables the assessment of individual internal metabolic stability, temporal fluctuations, and early deviations from homeostasis. Although the number of participants in the CHRIS project is not outstanding, CHRIS is also one of the important European resources for studying health baselines.

It is worth noting the Trinity Student Study (TSS) as one that may be considered to approach the analysis of a truly “healthy cohort”. TSS is a cross-sectional design study that aims to examine genotype-phenotype associations of blood metabolites in healthy young adults of an ethnically homogeneous background [150]. As part of a larger genetic-association study, a total of 3569 students initially applied, and of these students, 2524 individuals who had Irish grandparents and no major medical problems at the time of the study were invited to continue with the study and consented to give a venous blood sample [151]. Questionnaire data were collected from all participants, which included details on lifestyle choices, including smoking, alcohol intake (in grams of ethanol per day) and the use of contraceptives [150]. The advantage of the TSS cohort lies in the clear health status of the volunteer and the fact that the volunteers form a homogeneous cohort in terms of race and environment. This homogeneity reduces potential confounding factors, making the genetic association signals clearer. TSS has also reported many multi-omics results including metabolomics and genomics [150,151,152,153,154,155,156,157,158,159,160,161]. For instance, Brosnan et al. not only determined that the average serum formate concentration of 1701 healthy young people was 25.9 μM, but also found that in this group, the formate concentration was significantly positively correlated with one-carbon metabolism-related metabolites such as serine, methionine, tryptophan and choline, and negatively correlated with glycine and serum vitamin B-12 levels. The formate concentration of individuals with the TT genotype of the 5,10-methylenetetrahydrofolate reductase (MTHFR) 677C→T gene was lower than that of individuals with the CC genotype, indicating that the TT genotype of the MTHFR 677C→T gene may increase the risk of Neural Tube Defects (NTDs) [150]. In a separate study, Shane et al. demonstrated that the MTHFR 677C→T (rs1801133) variant was the major genetic modifier of all 3 folate-related biomarkers (erythrocyte folate, serum folate, and plasma total homocysteine) in the TSS, all reaching genome-wide significance. They also identified a second polymorphism in the MTHFR gene (rs3753584), the only MTHFR variant with an independently significant effect on erythrocyte folate, filling a research gap regarding folate [159]. TSS has created a unique control environment, allowing metabolic variations to be explained under the least interference from age, environment, socioeconomic background, or chronic diseases. Although TSS is a cross-sectional small-scale research group with a single background of volunteers, it provides one of the clearest examples of the metabolic profiles of healthy young people.

Although these population cohorts may approximate the majority of the conditions required for an ideal healthy population cohort, they provide unique controlled conditions. However, their single geographical conditions, limited sample size, and cross-sectional research methods limit their ability to capture the diversity at the population level. Larger and more diverse population cohorts are needed to create comprehensive metabolic baselines that span across gender, age, population, geographical environment, and biochemical methods. Currently, many large biobanks in various countries have begun to conduct in-depth metabolomics research, increasing in breadth and depth.

The UK Biobank project is a prospective cohort study that recruited approximately 500,000 UK residents between the ages of 40–69 years. Along with blood, urine and saliva fluid samples, health questionnaires and imaging data were collected. In addition, the dataset has a large amount of detailed genomic and phenotypic data, with more than approximately 90 million SNP marker loci. Thousands of studies have been completed using the Biobank database, including genetic studies, disease prediction, and biological target discovery, making Biobank one of the most important international resources for biomedical research [162,163,164,165,166]. In recent years, there have been numerous metabolomics research results related to various chronic diseases and cancers [167,168,169,170,171]. For instance, Bragg et al. utilized NMR metabolomics feature data to significantly enhance the predictive ability of the T2DM risk prediction model based on conventional risk factors [169]. Geng et al. identified 90 biomarkers that were significantly associated with the risk of incident chronic kidney disease(CKD), thereby enhancing the predictive ability for CKD risk [172]. Qiang et al. were the first to establish a metabolite-based risk score (MetRS), finding that specific metabolites (such as lipoproteins, glucose, and branched-chain amino acids) were significantly associated with the risk of dementia. When combined with demographic and cognitive indicators, the area under the curve (AUC) values for predicting all-cause dementia (ACD), Alzheimer’s disease (AD), and vascular dementia (VaD) reached 0.857, 0.861, and 0.873, respectively [173]. In 2023, Nightingale Health Plc. provided an NMR-based analysis conducted on over 120,000 participants, capturing 249 biomarkers related to small-molecule indicators such as lipoprotein lipids, fatty acids, amino acids, ketone bodies, and glycolytic metabolites, which were directly associated with over 700 common diseases, further enhancing its value in characterizing metabolic changes at the population level [174].

Involved in many of these national projects is Nightingale Health Plc., a large commercial supplier offering targeted metabolomics data for specific metabolic biomarkers and delivering a complete lab-to-data solution for human serum, plasma, or urine samples [175]. Nightingale Health’s technology has been applied to dozens of large global population cohorts, including the UK Biobank, Finnish National Biobank, Estonian Biobank, Mexico City Prospective Study, and South Asia Biobank, among others. It holds information on millions of samples, making it one of the world’s largest providers of standardized NMR-based metabolomics datasets. Its high-throughput ^1^H-NMR technology enables the absolute quantification of 250 biomarkers from a single blood sample, primarily covering lipoprotein subclasses, fatty acid composition, key amino acids, glycolysis-related metabolites, ketone bodies, and inflammation-associated markers. Buergel et al. employed the Nightingale platform to reveal reproducible associations between metabolic signatures and diseases including cardiovascular disorders, neurological conditions (such as dementia), and chronic metabolic diseases (such as T2DM) across diverse European population cohorts [176]. The standardized methodology employed by the same NMR platform enables cross-cohort comparisons between diverse populations, and such high comparability will undoubtedly also help define consistent metabolic baselines in the future. Although the number of biomarkers is significant (249 to be exact), one weakness of the Nightingale data is that only key metabolites are identified. For example, with regard to (i) glycolysis and related metabolites, glucose, lactate, pyruvate, citrate, and glycerol are the only ones quantified. Likewise, for (ii) ketone bodies, they are 3-hydroxybutyrate, acetate, acetoacetate, and acetone, for (iii) fluid balance and inflammation, they are creatinine, albumin, and glycoprotein acetyls, for (iv) amino acids, alanine, glutamine, glycine, histidine, isoleucine, leucine, valine, phenylalanine, tyrosine, and for (v) fatty acids, total fatty acids, omega-3, omega-6, polyunsaturated, monounsaturated, and saturated fatty acids, including specific measures like linoleic acid and docosahexaenoic acid (DHA) are covered. However, metabolites that are gaining increasing importance such as succinate and itaconate have not been identified amongst its list, and whether new compounds can be added post-analysis is not clear.

FinnGen is a large-scale biomedical research project from Finland, characterized by a relatively isolated genomic database of Finns. FinnGen collects biological samples, health data (including hospitalization records and medication records), and genome-wide genotyping data from approximately 500,000 Finns [177,178,179]. The project not only provides long-term comprehensive longitudinal data for Finland itself but also provides a unique and valuable resource for human disease research [180]. Currently, FinnGen, which has a large Genome-Wide Association Study GWAS database, mainly focuses on integrated research in genomics and metabolomics [181,182,183,184], delving deeply into the biological mechanisms of genes to diseases [181]. Kuriki et al. conducted a GWAS based on the FinnGen cohort and identified that a low-frequency variant located in an intron of TNRC18 increases susceptibility to inflammatory bowel disease (IBD); a frameshift mutation in MEPE markedly increases the risk of otosclerosis; and a missense variant in ANGPTL7 (p.Arg220Cys) lowers intraocular pressure and protects against glaucoma, thereby suggesting a novel therapeutic target [180]. FinnGen provides causal evidence for metabolite variations by achieving the integration of metabolic changes with gene associations and Mendelian randomization studies. Such multi-omics combined analysis is a powerful method for capturing the fundamental causes of standard health baseline deviations.

Apart from the Biobank in Europe and FinnGen, there are many other large biobanks around the world (Supplementary Table S1). For instance, (i) the “All of Us” research project initiated by the NIH in the United States has over 400,000 volunteers, 77% of whom are from historically underrepresented groups in biomedical research and 46% are individuals from under-represented racial and ethnic minorities [185]. (ii) LifeLines is a large, population-based, multidisciplinary dynamic cohort study and biobank which recruited 10% (approximately 167,000 people) of the population in the northern part of the Netherlands aged 0 to 93 years old [186,187]. In addition, (iii) the China Kadoorie Biobank (CKB) has recruited 500,000 volunteers from China, etc. [188].

Overall, these large biobanks provide extremely powerful data resources for health baseline research, as well as their diverse populations and high-quality longitudinal research characteristics. However, these databases were not established specifically for the purpose of studying health baselines. Factors such as metabolomics data from different platforms, complex population composition, and other issues cannot be solved in a short period of time. Therefore, further work is needed to integrate these resources and develop coordinated strategies that can reliably characterize metabolic homeostasis in a truly healthy state.

## 5. Conclusions

Metabolomics has emerged as a uniquely powerful approach for redefining how health is conceptualized, measured, and monitored at the molecular level. Unlike traditional clinical biomarkers, which typically reflect isolated physiological parameters, metabolomics captures the integrated downstream consequences of genomics, transcriptomics and proteomics, environmental exposure, lifestyle behaviors, and host–microbiome interactions. Even for individuals who provide ‘healthy control (HC)’ samples, metabolic profiles exhibit substantial and structured variability driven by age, sex, body mass index, physical activity, diet, microbiome composition, and population background. These findings challenge the long-standing notion of a single, static “normal range” for health and instead support a paradigm in which health is best understood as a dynamic, multidimensional state of biochemical equilibrium. It is in this context that the classical modus operandi of treatment was to eliminate/reduce the patient’s ailment or infirmity and return the patient to her/his previous state (not necessarily to a baseline of health).

A central insight emerging from current research is that defining a metabolic baseline of health cannot rely on cross-sectional population averages alone. While large cohort studies have been instrumental in mapping population-level metabolic distributions, they primarily provide snapshots rather than trajectories of metabolic homeostasis. Health, however, possesses a temporal dimension as well. Longitudinal metabolomic profiling is therefore essential to distinguish stable intra-individual variation from early pathological deviation. Repeated measurements within individuals allow the establishment of personalized metabolic reference ranges, against which subtle but meaningful perturbations can be detected well before clinical symptoms arise. In this context, time becomes as critical a dimension as the concentrations of metabolites (or the absence-presence-ratio profile of these metabolites) in defining a true health baseline.

At the same time, technical and analytical heterogeneity remains another obstacle to standardization. Differences between analytical platforms, particularly NMR- and MS-based metabolomics, limit cross-study comparability and complicate the establishment of universal reference frameworks. Rather than viewing these platforms as competing alternatives, the evidence strongly supports their integration. Multi-platform strategies, combined with advanced computational tools for metabolite identification and data harmonization, offer a path toward broader metabolome coverage and improved robustness. This is not simply a data management issue, as there are respective instrumentation-based, quality-control-related harmonization matters to be sorted as well. Moreover, the consistency in measurements and/or the equivalency of measured data points should be contextual, with age, gender, ethnicity, geography, and culture-influenced diet taken into account. Equally important is the recognition that metabolic baselines are biofluid and compartment specific, necessitating standardized protocols and metadata to ensure biological interpretability across tissues and sampling contexts.

It should be emphasized that the concept of a healthy metabolome is not the determination of a monolithic or generic set of data that can be universally applied. Rather, respective healthy metabolomes will align with the stratification of the wider population into sub-groups or clusters. Furthermore, with the continuous advancement of technology leading to further stratification, additional refinements of metabolomic health will continue to emerge and evolve.

In view of this, we suggest that during the exploratory phases, certain subgroups of the population that provide particularly clear metabolic signals could be initially tested. For example, samples from existing biobanks of individuals that have been extensively screened to be absent of diseases could be selected, albeit a labor-intensive task. Alternatively, it could be healthy young populations (such as the TSS mentioned above), elite athletes (such as members of national Olympic teams who have strictly regulated physiology through continuous training), or young adult personnel serving in the armed forces (with good cardiorespiratory fitness), etc. Although these groups do not represent the public, the relatively homogeneous nature of their diet and metabolic profiles can serve as the basis for a preliminary health baseline and then be applied to a wider range of populations for verification and expansion.

Without doubt, large-scale biobanks and population cohorts now provide unprecedented opportunities to address these challenges going forward. Although most were not originally designed to define health baselines, their scale, diversity, and increasing availability of multi-omics and longitudinal data make them valuable resources. The process is also naturally collaborative interdisciplinarily and internationally (and hence may require significant initial investment), but will bear fruit as a catalogue of multi-omic standards. When combined with artificial intelligence and systems-level data integration, these datasets can support the construction of stratified, context-aware metabolic reference models that accommodate both individual variability and population heterogeneity.

Ultimately, the goal of defining a metabolic baseline is not to replace clinical judgment but to enhance it. A robust, dynamic, and personalized metabolic framework has the potential to shift medicine from reactive disease treatment toward proactive health maintenance. By enabling earlier detection of deviation from physiological homeostasis, metabolomics-informed baselines can support risk prediction, targeted prevention, and individualized intervention strategies. As analytical technologies mature and longitudinal multi-omics data continue to accumulate, metabolomics will play a central role in molecularly defining health that reflects the full complexity of human physiology.

## Figures and Tables

**Table 1:** Key Differences Between NMR and MS Platforms (adapted from [82,100]).

	NMR	MS
Metabolite Coverage	200+ metabolites	100–1000 metabolites
Sensitivity	μM–mM range	pM–nM range
Sample Preparation	Relatively simple	(i) Requires extraction, sometimes multiple preparation steps, and (ii) Optimization of ionization conditions
Sample Recovery	Non-destructive, suitable for long-term storage.	Destructive, sample not recoverable, requires only small volumes.
Quantification	Absolute quantification possible due to the signal area-or-volume being directly proportional to the nuclei in a given environment; highly reproducible	Semi-quantitative; affected by batch effects
Target analysis	Inferior for targeted analysis	Superior for targeted analysis
Sample Measurement	All metabolites detectable observed in one measurement.	Requires different pre-MS treatments for different classes of metabolites

## Data Availability

No new data were created or analyzed in this study.

## Supplementary Materials

The following supporting information can be downloaded at: https://www.mdpi.com/article/10.3390/biom16050683/s1, Table S1: Representative molecular studies of human health. Refs. [189,190,191,192,193,194,195,196,197,198,199,200,201,202,203,204,205,206,207,208,209,210,211,212,213,214,215,216,217,218,219,220,221,222,223,224,225,226,227,228,229,230,231,232,233,234,235,236,237,238,239,240,241,242,243,244,245,246] are cited in Supplementary Materials.

## Abbreviations

3-Met-His	3-methylhistidine
AC	African-Caribbean
ACD	all-cause dementia
ACSM2A	Middle-Chain Acyl-CoA Synthetase 2A
AD	Alzheimer’s disease
AI	artificial intelligence
ANGPTL7	Angiopoietin-related protein 7
AUC	area under the curve
BCAA	Branched-chain amino acid
BMI	Body mass index
CE-MS	capillary electrophoresis-mass spectrometry
CLIR	Collaborative Laboratory Integrated Reports
C0	free carnitine
CHRIS	The Cooperative Health Research in South Tyrol
CKB	The China Kadoorie Biobank
CKD	chronic kidney disease
COPD	chronic obstructive pulmonary disease
DASH	Dietary Approaches to Stop Hypertension
DHA	docosahexaenoic acid
ETFDH	Electron Transfer Flavoprotein Dehydrogenase
ExWAS	Exome-wide association analysis
FDA 510(k)	US Food & Drug Administration 510(k); A premarket submission made to FDA to demonstrate that the device to be marketed is safe and effective
fm	femtomoles
GABA	gamma-aminobutyric acid
GC-MS	Gas Chromatography-Mass Spectrometry
GFR	glomerular filtration rate
GLYAT	Glycine N-acyltransferase-like 2
HC	healthy control
HDL	High-density lipoprotein
hsCRP	high-sensitivity C-reactive protein
hURV	Hierarchical Remove of Unwanted Variation
IBD	inflammatory bowel disease
KarMeN	Karlsruhe Metabolomics and Nutrition
LC-MS	Liquid Chromatography-Mass Spectrometry
MCCC2	Methylcrotonyl-CoA carboxylase subunit 2
MetRS	metabolite-based risk score
ML	Machine learning
MS	Mass-Spectrometry
MTHFR	Methylenetetrahydrofolate reductase
NMR	Nuclear Magnetic Resonance
NTDs	Neural Tube Defects
PA	Physical activity
PBHS	The Project Baseline Health Study
pm	picomoles
PRS	polygenic risk scores
PUFAs	Polyunsaturated fatty acid
QC	Quality control
RSD	Relative standard deviation
SA	South Asian
SERRF	Systematic Error Removal using Random Forest
T2DM	Type 2 Diabetes
TMAO	trimethylamine-N-oxide
TMM	The Tohoku Medical Megabank Project
TNRC18	Trinucleotide Repeat Containing 18
TSH	thyroid-stimulating hormone
TSS	The Trinity Student Study
Vad	vascular dementia
WE	White European
WHO	World Health Organization
