# Supplementary material for: Metabolomics and Human Health: Progress, Insights, Challenges and the Concept of a Healthy Metabolome
Source: Biomolecules. 2026 May 5;16(5):683. doi: 10.3390/biom16050683 (PMC13204543; doi:10.3390/biom16050683)
Supplement: Supplementary file 1 [file biomolecules-16-00683-s001.zip › biomolecules-4112102-supplementary.pdf]

Supplementary Table. Representative molecular studies of human health

Name of study (Starting Year)	Cohort samples	Objectives	Key Publications	Constraints	Key advantages	Methodology
The Nurses' Health Study (NHS) (1976)	121 700 married registered nurses aged 30-55 years old.	To update exposure assessment for a broad range of lifestyle factors, endogenous hormones and DNA, in relation to risk of chronic diseases.	[189–193]	Restricted to female healthcare professionals and not designed as a health-enriched cohort, with limited ethnic diversity and lack of systematic metabolomic profiling, which limits its suitability for defining a generalizable metabolic health baseline.	A large, well-established prospective cohort with decades of follow-up, detailed lifestyle and dietary assessments, and repeated health outcome collection, providing a valuable framework for studying long-term metabolic variation across adult life stages.	A large prospective cohort study initiated in 1976, enrolling female registered nurses in the United States and following them through biennial questionnaires to collect detailed information on lifestyle, diet, medication use, and health outcomes. The study includes long-term follow-up with validated disease ascertainment and repeated collection of biospecimens, enabling longitudinal epidemiological and molecular analyses
Health Professionals Follow-Up Study (HPFS) (1986)	51,529 U.S. male health professionals aged 40-75 years old.	To evaluate hypotheses about men's health related to diet and lifestyle to the incidence of serious illnesses, such as cancer, heart disease, and other vascular diseases.	[194–198]	Male-only, occupation-specific cohort not health-enriched by design, limiting generalizability for defining a universal metabolic health baseline.	Long-term prospective cohort with high-quality lifestyle data and biospecimens, enabling longitudinal assessment of metabolic variation.	Participants are followed through biennial questionnaires to update lifestyle, medical history, and health outcomes, with detailed dietary assessments administered every four years. Biospecimens including blood, DNA, and tissue samples are collected to support molecular and epidemiological analyses.
Women's Health Initiative (WHI) (1991)	161808 postmenopausal women aged 50 - 95+ y in the United States.	A long-term national health study that focuse+20:22s on strategies for preventing heart disease, breast and colorectal cancer, and osteoporosis in postmenopausal women. These chronic diseases are the major causes of death, disability, and frailty in older women of all races and backgrounds.	[199–202]	Restricted to postmenopausal women aged 50–79 and not health-enriched by design, with metabolomic analyses conducted mainly in subcohorts rather than through systematic, repeated profiling of the full cohort, limiting definition of a universal or dynamic metabolic baseline.	A very large, multicenter prospective cohort of over 161,000 postmenopausal women with long-term follow-up, standardized data collection, and extensive stored biospecimens, providing robust population-level reference data for mid-to-late-life female health.	A large multicenter study began in the early 1990s and recruited over 161,000 postmenopausal women aged 50-79 years in the United States, including randomized clinical trials and observational studies. Participants underwent standardized baseline assessments and long-term follow-ups through regular questionnaires, clinical measurements, and biological specimen collection to evaluate lifestyle factors, health outcomes, and disease risks.
Twins UK (1992)	~12,000 volunteer adult twins from across the UK (aged 18–103 years), predominantly female.	To investigate genetic and environmental determinants of healthy ageing by longitudinally tracking physiological changes across multiple organ systems using twin cohorts and integrated omics data.	[203–207]	Volunteer twin cohort with limited representativeness, heterogeneous baseline measures, and incomplete longitudinal harmonization, constraining generalization and precise definition of a standardized metabolic health baseline.	Twin-based studies with long-term, standardized and repeated phenotyping allow clearer separation of genetic and environmental influences on metabolic variation, helping to define normal physiological variability and near-baseline health states.	Through multiple home visits, repeated questionnaire survey data and standardized clinical phenotypes were collected, and multiple biological specimens (such as blood, urine, feces, saliva and some tissues) were stored for multi-omics analysis (genotyping/sequencing, epigenetics, transcriptomics, metabolomics, microbiomics) and linked with cancer and mortality records for follow-up.
Dietary Approaches to Stop Hypertension trial (DASH) (1997)	456 healthy men and women, aged 22 years or older, with systolic blood pressure less than 160 mm Hg and diastolic blood pressure 80 to 95 mm Hg.	To evaluate the effects of predefined dietary patterns on blood pressure under controlled feeding conditions in healthy adults.	[71,208,209]	Short intervention duration, small sample size, cross-sectional design, and limited phenotypic and molecular data collection restrict its relevance for defining long-term metabolic health baselines.	Highly controlled dietary intervention in metabolically stable, generally healthy adults, providing a clean experimental setting to characterize short-term metabolic responses under near-ideal nutritional conditions.	Participants first completed a 3-week run-in on the control diet, then were randomized to one of three dietary patterns (control, fruits-and-vegetables, or "ideal" DASH diet) for an 8-week intervention; all meals were provided, body weight and sodium were kept constant, and blood pressure was repeatedly measured (primary endpoint: change in diastolic BP from baseline to end of intervention).
Trinity Student Study (TSS) (2003)	2,524 healthy young Irish adults aged 18–28 years of Irish ancestry.	Study genotype–metabolite associations under optimal health.	[150–153,155]	Limited age range, small sample size, and restricted ethnic and lifestyle diversity.	Highly homogeneous cohort of rigorously defined healthy young adults, minimizing disease- and lifestyle-related confounding for baseline characterization.	Healthy college students aged 18-28 years old, without major diseases and of Irish descent, were recruited. Participants provided fasting blood samples of serum, EDTA plasma, and red blood cell lysates, as well as detailed lifestyle questionnaires. The samples were processed within 3 hours of collection and stored at -80°C for downstream metabolomics and genetic analysis.

Name of study (Starting Year)	Cohort samples	Objectives	Key Publications	Constraints	Key advantages	Methodology
China Kadoorie Biobank (CKB) (2004)	More than 512,000 adults were recruited from ten geographically defined and diverse areas of China.	To assess the roles of both established and emerging risk factors in aetiology of a wide range chronic diseases;<br>To determine the complex interplay between genes and lifestyle and environmental factors and between different genes on the risks of major chronic diseases;<br>To explore and identify novel biological mechanisms linking particular risk factors with different diseases and to inform development of new treatments.	[210–214]	Study design omits observation of young people (teenagers and young adults) which may have provided capturing insights into phenotypic changes from younger ages.	A large-scale prospective cohort study with long-term follow-up, along with high-quality biological sample collection, can characterize the metabolic distribution and trajectory at the population level in different environments.	Collect standardized baseline questionnaire data, physical measurements and blood samples, and conduct on-site tests in the long-term biobank. Then, conduct regular surveys on approximately 5% of the surviving participants (in 2008, 2013-2014, and 2020-2021) and determine the results through nearly complete electronic connections with death and disease registries as well as the national health insurance system (hospital events). In necessary cases, active follow-ups will be conducted as well.
The Long Life Family Study (2005)	5,097 participants (1,704 proband generation aged over 78 y, and 3,393 offspring generation).	To create and enroll a cohort of families enriched for exceptional longevity (EL) in order to discover factors that contribute to healthy aging and survival.	[215–217]	Predominantly elderly and longevity-enriched participants limit generalizability to younger populations and constrain its use for defining a universal metabolic health baseline.	Enrichment for long-lived individuals with preserved functional health provides a unique opportunity to characterize physiological variation associated with healthy ageing.	A family-based cohort study enrolling long-lived individuals and their offspring, using standardized in-person baseline examinations, detailed phenotyping, fasting blood biomarker collection, and prospective follow-up to study genetic and environmental determinants of healthy ageing and longevity.
METabolic Syndrome In Men Study (METSIM) (2005)	10,197 Finnish men (aged 45 -74), randomly selected from the population register of Kuopio, Eastern Finland (population 105,000).	To investigate nongenetic and genetic factors associated with the risk of T2D and CVD, and with cardiovascular risk factors.	[218–221]	Male-only, predominantly middle-aged cohort enriched for metabolic risk, with limited age, sex, and ethnic diversity, restricting its suitability for defining a generalizable metabolic health baseline.	All participants have standardized metabolomic profiling and genome-wide genetic data.	This study collects detailed data on cardiac metabolic risk factors through questionnaire surveys and standardized clinical measurements (such as anthropometry, blood pressure, and body composition), aiming to study the genetic and non-genetic determinants of T2D/CVD/insulin resistance characteristics in cross-sectional and follow-up settings.
UK Biobank (2006)	Approximately 500,000 UK residents aged of 40-69 years.	To improve the prevention, diagnosis, and treatment of serious and chronic diseases in a large population and enabling open-access biomedical research worldwide.	[162,174,222,223]	A predominantly middle-aged to older adult population and the lack of systematic longitudinal metabolomic measurements limit its suitability for defining a precise metabolic health baseline.	Large-scale population cohort with standardized NMR-based metabolomics (e.g., Nightingale Health platform) and rich phenotypic data, providing strong statistical power to map population metabolic distributions.	Participants completed standardized baseline touchscreen questionnaires and interviews (including socio-demographic information, lifestyle, diet, smoking/alcohol consumption, medical history, etc.), underwent physical measurements (such as blood pressure, anthropometric measurements, grip strength, lung function, vision), and provided blood and urine samples for long-term storage and large-scale analysis. Additional evaluations were conducted on subsets (such as imaging, accelerated measurements, online questionnaires, repeated assessments) and long-term follow-up was carried out through linkage. National health records (cancer and death registration, hospitalization information, and available primary care data).
Lifelines Biobank (2006)	Over 167,000 participants from the northern Netherlands, including three generations within families.	To generate deep pheno- and genotypic data to enable research on the development of chronic and rare diseases and their risk factors with the ultimate goal to contribute to healthy ageing, public health and precision medicine.	[224–227]	Selective attrition across follow-up waves and underrepresentation of ethnic minorities may bias longitudinal estimates and limit the generalizability of metabolic health baselines.	Large and stable longitudinal cohort with repeated phenotyping, rich biological samples, and a multigenerational structure, providing a strong foundation for capturing normal physiological variation and defining population-level health baselines.	Through extensive questionnaire surveys and standardized physical examinations conducted at the research site, baseline data of 167,729 participants (aged from 6 months to 93 years) were collected. Participants were surveyed every 1.5 years and underwent research-site examinations every 5 years. Fasting blood and 24-hour urine samples were collected on the same day for processing and were long-term stored in the biobank; connections with medical registries and environmental data are being established.

Name of study (Starting Year)	Cohort samples	Objectives	Key Publications	Constraints	Key advantages	Methodology
The Million Veteran Program (MVP) (2011)	More than one million veterans from the United States.	To examine genetic and nongenetic determinants of chronic diseases.	[228–232]	Predominantly cross-sectional survey data, incomplete self-reported information, and limited repeated longitudinal assessments; cohort restricted to U.S. veterans, potentially limiting generalizability to the broader population.	Large, ethnically diverse cohort with broad age coverage and growing metabolomics datasets; the relatively high baseline physical fitness of veterans offers a useful reference for studying metabolic variation around near-healthy states at population scale.	MVP combines questionnaires (Baseline + Lifestyle), linkage to VA electronic health records and administrative databases, and a blood draw that is processed and stored (plasma/buffy coat/DNA) for genotyping and sequencing (with plans for additional omics).
Cooperative Health Research in South Tyrol (CHRIS) (2011)	13,393 adults aged ≥18 years from the Vinschgau region (median age 46 years; range 18–94), with balanced sex distribution and high local representativeness.	To investigate genetic, molecular, behavioral, and environmental determinants of aging-related cardiovascular, metabolic, and neuropsychiatric conditions through a population-based, longitudinal cohort.	[145–149]	Potential selection bias due to prolonged recruitment periods and intensive data collection; moderate sample size compared with national biobanks.	Deep repeated phenotyping with integrated genotype and multi-omics data in a stable, high–healthy-life-expectancy population, enabling characterization of near-baseline physiological variation.	Participants have an in-person baseline visit with overnight fasting, standardized clinical exams (blood/urine biochemistry, anthropometrics, BP, ECG, tremor assessment), computer-assisted interviews and questionnaires on lifestyle/exposures, and extensive biobanking (blood fractions, urine, DNA) with genotyping and LC–MS metabolomics.
The Karlsruhe Metabolomics and Nutrition Study (KarMeN) (2012)	301 healthy adults, 172 men, 129 women, aged 18–80 years old, BMI 17.8–31.4 kg/m^2	A cross-sectional study to investigate the inherent variation in the human metabolome including healthy male and female participants spanning a wide age range.	[47,48,233–235]	Limited sample size, cross-sectional design, and recruitment from a single geographic region restrict generalizability; lack of longitudinal follow-up prevents assessment of intra-individual metabolic stability over time.	KarMeN focuses on strictly selected healthy adults across a wide age range and applies highly standardized, multi-platform metabolomics (NMR, GC-MS, LC-MS) in blood and urine, providing a well-controlled reference framework for defining physiological metabolic baselines.	Participants completed multiple standardized study visits within a defined time window, including detailed anthropometric, clinical, functional, dietary, and physical activity assessments. Fasting blood samples and 24-hour and spot urine samples were collected under strictly controlled protocols and analyzed using a multi-platform metabolomics approach combining ^1H NMR, GC-MS, and LC-MS.
Tohoku Medical Megabank (TMM) (2012)	TMM CommCohort Study: Approx. 84,000 Residents of Miyagi Prefecture and Iwate Prefecture who are 20-years of age and older.<br>TMM BirThree Cohort Study: Approximately 73,000 participants, comprising ~41,000 adults (pregnant women living in Miyagi Prefecture and their relatives) and ~32,000 children (newborn infants and their siblings).	Study the long-term effects of the Great East Japan Earthquake on health, and by integrating biological resource databases, including genomic and metabolomic data, clarify the genetic, environmental and lifestyle determinants of major chronic diseases.	[134,135,139,236,237]	Geographically restricted cohort; may be difficult to build a standardized metabolic health baseline.	Large-scale prospective population-based cohorts with integrated genomic and metabolomic profiling and rich lifestyle data, allowing long-term evaluation of gene–environment and lifestyle interactions.	Residents are recruited through municipal health checkups, community support centers, and collaborating obstetric clinics and hospitals. Standardized questionnaires, clinical and physiological measurements, and biological specimens (including blood and urine from all participants, and additional samples such as saliva and umbilical cord blood) are collected and stored in an integrated biobank, with follow-up conducted via repeated surveys, health examinations, and linkage to health records and registries.
The Enable Study (2016)	503 healthy volunteers including 44 3- to 5-year-old children, 94 young adults aged 18–25 years, 205 adults aged 40–65 years ("middle agers") and 160 older persons aged 75–85 years.	To investigate age-specific dietary patterns and nutritional needs, and their associations with metabolic and clinical health indicators. Such insights provide a basis for understanding how dietary factors contribute to health across the life course and may inform age-related nutritional recommendations.	[45,238]	Limited sample size, ethnically and geographically homogeneous cohort, and cross-sectional study design.	Strictly selected healthy participants across 4 distinct life stages, combined with highly standardized deep phenotyping, provide a clean reference framework for age-specific physiological and metabolic baseline states.	The participants underwent standardized and comprehensive phenotypic analysis following strict standard operating procedures, which included anthropometric measurements, body composition, energy metabolism, metabolic tests, dietary assessment, lifestyle questionnaire, and biological sample collection (blood, urine, feces, saliva).

Name of study (Starting Year)	Cohort samples	Objectives	Key Publications	Constraints	Key advantages	Methodology
Project Baseline Health Study (PBHS) (2017)	Participants are drawn from the registry to cover the full spectrum of health, from exceptional health to diagnosed disease.	(i) develop a set of scalable and standardized tools and technologies to collect, organize, and analyze clinical, molecular, imaging, sensor, self-reported, behavioral, psychological, environmental, and other health-related measurements; (ii) evaluate the use of sensor technologies for the collection of more continuous, accurate health information; (iii) create a dataset encompassing a wide spectrum of phenotypic measures; (iv) measure the phenotypic diversity observed among a participant population and its trajectory in health and disease; (v) share data with qualified investigators to extend learning and create an example of open science.	[5,41,42,239]	Not specifically designed to define a metabolic health baseline; Comprehensive data collection, however studies such as the systematic integration of metabolic profiles in a healthy state are not specified.	Deep, longitudinal multi-dimensional phenotyping integrating clinical, molecular, imaging, sensor, and behavioral data.	Self-reporting, general practitioner medical, molecular, imaging, sensor-based, behav-ioral and psychological measurements. For the molecular measurements, lipids, high-sensitivity C-reactive protein (hsCRP), creatinine, ions, serum proteins, glomerular filtration rate (GFR), hematology, thyroid stimulating hormone (TSH) and others.
FinnGen (2017)	More than 520,000 participants (aged 0~ 90 + years old) from all over Finland.	To establish a unique large-scale genomic and health resource database, aim to enhance our understanding of the genetic background of diseases, as well as to facilitate drug development and the application of genomic medicine in clinical practice.	[180,240–242]	Greater focus on GWAS.	A large-scale genetic cohort with integrated metabolomics can conduct a powerful analysis of the genetic determinants of metabolic variations in specific populations and support causal inferences between metabolites and disease risk.	A national biobank registry study, using the consented samples from the Finnish Biobank Network, links genotypes with longitudinal national health registries (such as hospital diagnoses, drug reimbursements, cancer and death registrations), and conducts whole-genome genotyping and imputation to conduct large-scale association analyses for thousands of disease endpoints.
All of US (2018)	Over 400,000 volunteers from United States, 77% of whom are from historically underrepresented groups in biomedical research and 46% are individuals from under-represented racial and ethnic minorities.	To build a large, diverse population cohort with integrated genomic, clinical, survey, and digital health data to enable broadly generalizable biomedical research and precision medicine.	[243–246]	Not a health enriched cohort and lacking sufficient longitudinal metabolomic dataset.	Designed to meet established diversity standards in health disparities research, with recruitment targets of over 45% racial and ethnic minority participants and more than 75% from historically underrepresented groups.	Data collection involves standardized health surveys, electronic health records, physical measurements, biological specimen collection (blood and urine), and optional digital health data, as well as continuous longitudinal tracking and centralized data coordination, to support large-scale observations and molecular analyses.

The studies have been identified and compiled through a structured and transparent search to minimize selection bias. Relevant studies were retrieved from PubMed, Web of Science, and Google Scholar using combinations of keywords including "metabolomics," "metabolic profiling," "health," "healthy baseline," "physiological homeostasis," and "population cohort." Priority was given to large-scale human cohort studies, representative methodological papers, and recent reviews that address metabolic variation in apparently healthy populations. This approach was informed by the general principles of the PRISMA guidelines, while allowing flexibility appropriate for a conceptual and integrative review.
